# World Health Organization repository of systematic reviews on interventions in environment, climate change and health: a new resource for decision makers, intervention implementers, and researchers

**DOI:** 10.1186/s12940-024-01105-y

**Published:** 2024-10-22

**Authors:** Shreya Shrikhande, Jennyfer Wolf, Cristina Vert, Alexandra Egorova, Maria Neira, Annette Prüss

**Affiliations:** 1 Swiss Tropical and Public Health Institute, Allschwil, Switzerland; 2 University of Basel, Basel, Switzerland; 3 Department of Environment, Climate Change and Health, World Health Organization, Avenue Appia 20, Geneva, 1202 Switzerland

**Keywords:** Interventions, Environment, Health, Systematic reviews, Policy-making, Overview, Climate change, Environmental health, Policies, Evidence

## Abstract

To facilitate the use of the mounting evidence on how human health is inextricably linked to the health of the planet and the urgent need for measures against the escalating triple planetary crisis, the WHO has developed a repository of systematic reviews on interventions in the area of environment, climate change and health (ECH). This commentary introduces the repository, describes its rationale and development, and points to potential future evolutions. The repository aims to provide a user-friendly tool for quickly finding systematic reviews and meta-analyses on specific ECH topics. The spreadsheet includes details on each systematic review, such as population, intervention type, control group, outcomes, and location, among other information. This supports effective assessment of the available evidence, potentially informing policy decisions across various sectors. The repository is a resource for anyone interested in the interlinkages between health and environment and is also targeted at decision makers, intervention implementers and researchers in order to identify priority issues and support evidence-based action. Furthermore, it can be used to identify areas in need of greater research. Additionally, systematic reviews of intervention effectiveness are often used for setting general guidelines and standards, for choosing the most promising intervention in a certain situation and for calculating the disease burden attributable to a specific environmental risk.

## Background

The world is facing a triple planetary crisis, marked by escalating climate change, alarming biodiversity loss, and a significant increase in environmental pollution, all of which have a critical impact on human health. It was estimated that in the year 2016 as much as 24% of global human deaths were attributable to environmental risks [1], which could be largely prevented through healthier environments.

Safely managing water and sanitation, maintaining indoor and outdoor air quality, and implementing climate change mitigation and adaptation measures are some of the strategies for healthier environments. Similarly important are the measures for managing chemicals and radiation, handling solid waste effectively, reducing noise pollution, and enhancing occupational health and safety. Ensuring safe and active mobility, protecting nature and biodiversity, and providing sustainable, healthy diets are also crucial elements for safeguarding human health [2].

Reducing environmental degradation and pollution is now widely recognized to have profound positive impacts on health [3]. The evidence on interventions in environment, climate change and health (ECH) has however not yet been systematically compiled and made available to decision makers. This gap makes it more difficult for policy makers and practitioners from various sectors to know about and choose the most effective interventions. Recognizing this critical need, the World Health Organization (WHO) developed the first repository of systematic reviews on interventions in environment, climate change and health, which can be accessed and downloaded from the following reference [4]. The reviews included address interventions in all major ECH topics with the intention of improving health or reducing harmful environmental exposures.

## Methods

We used the Cochrane guidance for conducting an overview of systematic reviews [5]. A protocol for this research was defined prior to the searches and can be obtained from the corresponding author.

For the repository, ECH risks and health topics are consistent with those outlined in the Compendium of WHO and other UN guidance on health and environment [2] and include the following: (i.) air pollution, including ambient and indoor/household air pollution, second hand tobacco smoke, dampness and mould; (ii.) water, sanitation and hygiene (WASH), including unsafe drinking water, inadequate sanitation, inadequate personal hygiene, and unsafe recreational water usage; (iii.) health risks from climate change; (iv.) exposure to solid waste, including e-waste and other hazardous waste; (v.) hazardous chemicals; (vi.) radiation, including radon, other x-rays and UV-radiation; (vii.) nature and health, including health risks from altered vector breeding, changes in ecosystems and biodiversity and exposure to nature; (viii.) safe environments and mobility, including health risks linked to the built environment, unsafe environments increasing the risk for unintentional injuries (such as drownings, burns and falls); (ix.) unsafe, unhealthy and unsustainable food; (x.) excessive noise; (xi.) priority settings for action, including unsafe and inadequate housing, unsafe and unhealthy workplaces, unsafe cities and urban settlements and unsafe, unsustainable health care facilities; and (xii.) cross-cutting topics in environmental health. We acknowledge that several of these topics are overlapping, such as climate change and air pollution. Research included in the repository can therefore be listed in more than one section. The final sheet of the repository excel file includes the complete list of included reviews.

We searched PubMed and Scopus using both Medical Subject Heading (MeSH) terms and keywords. MeSH terms are controlled vocabulary for indexing and searching biomedical literature, whereas keywords are freely chosen words or phrases on a certain topic. We also searched the Cochrane webpage on reviews about occupational safety and health [6], which provides a comprehensive list of Cochrane reviews on this topic. The search includes systematic reviews or overviews of systematic reviews on interventions covering a period from 1 January 2004 to 8 May 2023. We conducted separate searches for each of the ECH topics and sub-topics to allow for tailoring of the searches including adapting the search terms to the respective ECH topic, interventions, outcomes, and the type of study. The search terms can be downloaded from the WHO webpage [4]. Eligible reviews needed to be published in a peer-reviewed scientific journal in English, and to examine intervention’s effectiveness through either changes in health or exposure status. Systematic reviews considering exclusively medical and pharmaceutical, and other non-ECH interventions were not included. No further restrictions on intervention type, location or population subgroups were applied. Given the heterogeneity in certain ECH topics, we made some small adaptions of inclusion and exclusion criteria to certain topics, details for these are described in the tab “ECH topics” of the repository [4].

Identified records from the individual searches were imported to Endnote for de-duplication. As there were significant overlaps between different ECH topics, all identified records were pooled for screening. One reviewer (S.S.) screened study titles, abstracts and full texts and extracted the data of the systematic reviews that were chosen for inclusion. A second reviewer (J.W.) was consulted in case of doubt over the inclusion of a particular systematic review. Differences between the reviewers were reconciled through a third reviewer (A.P.).

We extracted data to a systematic form following the PICO framework to include information on the population, interventions, controls and health and/or exposure outcomes (including statistics if available) [7]. We also extracted information on title, authors, year of publication, study design (systematic review or meta-analysis), name of journal, access link (URL link/DOI etc.), ECH topic(s), and study location or setting.

## Findings

A total of 71,514 records were identified from the search of the databases. After removing duplicates, 35,550 records were screened by title and abstract, and 1,250 reviews were chosen for full text review. 976 individual records were included in the repository, further categorized within 12 main ECH topic and 38 sub-topics (Table 1).

**Table 1:** Number of systematic reviews on ECH interventions included for each topic and subtopic

ECH Topic	ECH Sub-topic	No. reviews
Air pollution	Ambient air pollution	9
	Indoor/household air pollution	25
	Second hand tobacco smoke	25
	Dampness and mould	1
Water, sanitation and hygiene (WaSH)	Water: unsafe drinking water	8
	Water: unsafe recreational water usage	9
	Inadequate sanitation	21
	Inadequate personal hygiene	40
	WaSH combined	28
Climate change	High/low Temperature	11
	Other climate risks	34
	Greenhouse Gases	15
Solid waste	Hazardous waste	1
	E-waste	1
	Household waste	4
	Microplastics	0
Chemicals	Hazardous chemicals	16
	Chemical incidents	0
Radiation	UV - natural and artificial	20
	Electromagnetic fields (EMF)	0
	Radiation exposure in healthcare	4
	Radon	1
	Radioactivity in food and drinking water	0
	Radiological emergencies	0
Nature and health	Changes in ecosystems and biodiversity	79
	Nature exposure	73
	Altered vector breeding and distributions	45
Safe environments and mobility	Unsafe, unhealthy and non-sustainable transport and mobility	72
	Unsafe environments increasing the risk for unintentional injuries (including injuries from drowning, burns, falls)	76
	Built environment	25
Safe and healthy food	Unsafe, unhealthy and unsustainable food	87
Environmental noise	Excessive environmental noise	11
Priority settings for action	Unsafe cities and urban settlements	21
	Unsafe and inadequate housing	34
	Unsafe and unhealthy workplaces	234
	Unsafe health care facilities	42
Cross-cutting topics	Children’s environmental health	37
	Environmental health	12
**Total**	**ECH interventions**	976*

* As one review can be included in more than one ECH subtopic, the sum of the number of reviews in Table 1 is higher than the total number of individual ECH reviews included in the repository. ECH: environment, climate change and health; WASH: water, sanitation and hygiene, e-waste: electronic and electrical waste

**Fig. 1:**
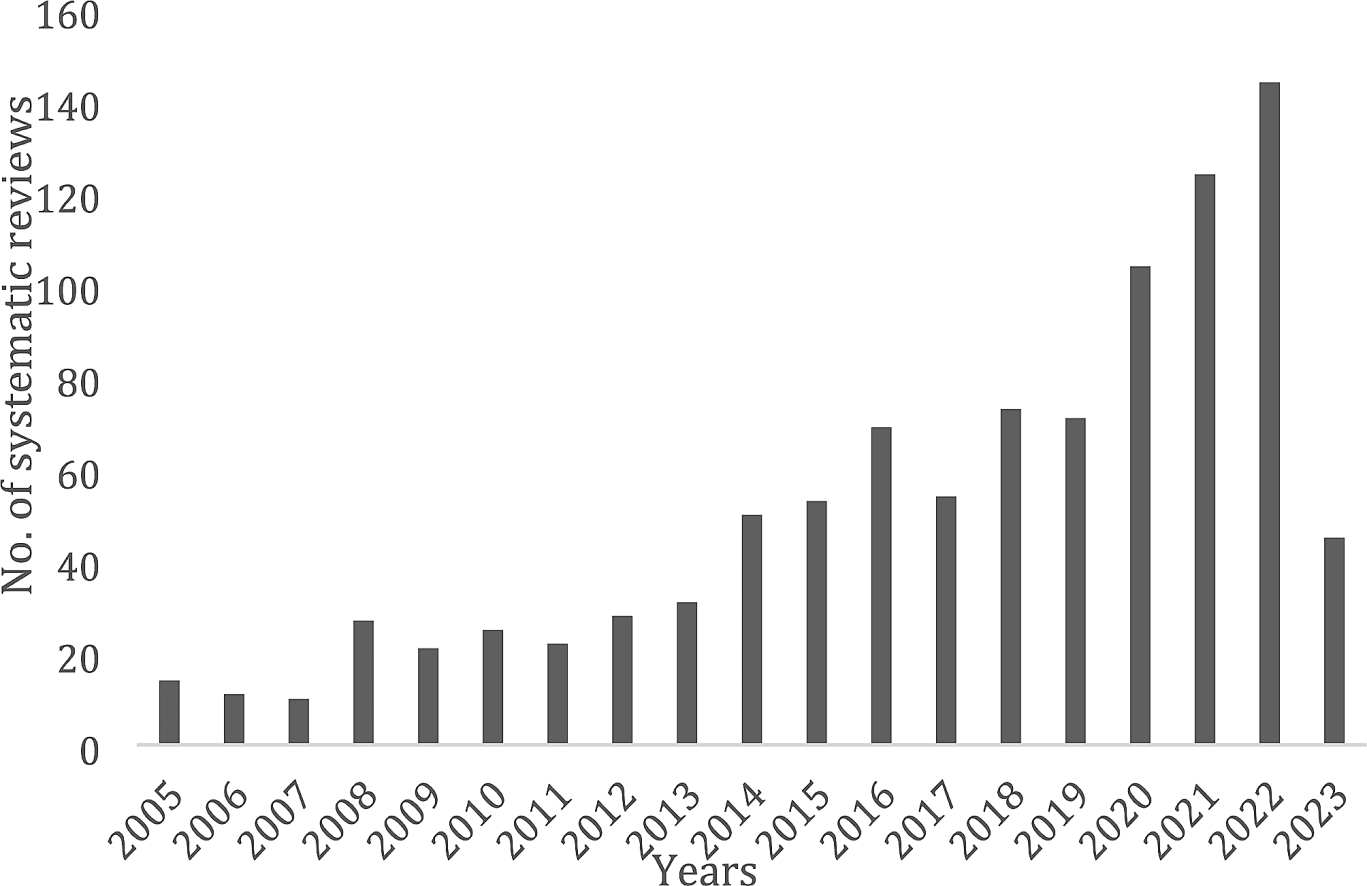
Number of systematic reviews on ECH interventions by year from 2005 to May 2023

There was a steep increase over time in the number of published systematic reviews on ECH interventions. We identified 14 systematic reviews on ECH interventions in 2005 and 144 in 2022 (Fig. 1).

While we identified many systematic reviews for major ECH topics (such as WASH and air pollution), we did not identify any systematic review on some subtopics, such as on micro-plastics, chemical incidents, electromagnetic radiation, radio activity in food and water, and radiological emergencies, and only a single review on dampness and mould, hazardous waste, e-waste, and radon respectively (Table 1).

## Discussion

We introduce the first version of the WHO repository of systematic reviews of ECH interventions which is based on an overview of reviews spanning a large range of environmental health topics. The systematic reviews included in the repository examine a broad range of ECH interventions, ranging from policies and practices to educational and awareness raising campaigns.

This tool aims at supporting policy makers, intervention implementers, including health system planners and administrators, health care practitioners, or public health officials, researchers and other actors by allowing them to more easily assess the available evidence on ECH interventions. This is especially important as the field of environmental health research in interventions effectiveness is rapidly expanding. The repository can inform policy decisions, as well as advocacy campaigns, aimed at tackling environmental health challenges. It may also assist in identifying ECH areas in need of additional research and inform exposure-response relationships for environmental burden of disease and related assessments.

The repository is a ‘live’ tool and is planned to be regularly updated. We are looking forward to developing collaborations with the environmental health community to keep the repository up-to-date and learn about user experiences and additional evidence (please contact us at ebdassessment@who.int). This valuable information will provide insights into areas requiring enhancement, support the identification of effective strategies, and highlight limitations, among other aspects. This is key to continually refining and improving the repository, and to ensuring a relevant, up-to-date, and rigorous source of information.

Future developments of the repository may include additional information for each included systematic review, such as plain language summaries that also pinpoint major research gaps, assessments of the methodological quality of included systematic reviews [8], and the certainty of evidence [9] and listing more detailed statistical measures, such as the relative risk for the health outcome for each review. These may also include measures for compliance, behaviour change, and other relevant information on intervention implementation.

## Conclusions

The WHO repository of systematic reviews on interventions in environment, climate change and health offers an extensive compilation of systematic reviews of interventions covering all major ECH areas. The repository equips policy makers and other actors with a tool that provides an overview of the available evidence on the effectiveness of ECH interventions, which can support them to select evidence-based interventions and strategies to protect health from environmental risks and to create healthier environments.

**Table:** 

The repository of systematic reviews on interventions in environment, climate change and health can be downloaded from this page: https://www.who.int/publications/m/item/repository-of-systematic-reviews-on-interventions-in-environment--climate-change-and-health. We are seeking academic collaborations for keeping the repository up-to-date (self-funded) in order to support evidence-based prevention in this area. For further information, please contact ebdassessment@who.int.	

## Data Availability

The search strategy and datasets generated during the current study are available in the ‘World Health Organization repository of systematic reviews on interventions in environment, climate change and health’,https://www.who.int/publications/m/item/repository-of-systematic-reviews-on-interventions-in-environment--climate-change-and-health

## References

[1] Prüss-Ustün A, Wolf J, Corvalán C, Bos R, Neira M. Preventing disease through healthy environments: a global assessment of the environmental burden of disease from environmental risks. Geneva, Switzerland: World Health Organization; 2016.

[2] Compendium of WHO and other UN guidance on health and environment: 2024 update [Internet]. Geneva: World Health Organization, United Nations Development Programme (UNDP), United Nations Environment Programme (UNEP) & United Nations Children’s Fund (UNICEF). 2024. https://www.who.int/publications/i/item/9789240095380.

[3] Landrigan PJ, Fuller R, Acosta NJ, Adeyi O, Arnold R, Baldé AB, et al. The Lancet Commission on pollution and health. Lancet. 2018;391:462–512. 29056410 10.1016/S0140-6736(17)32345-0

[4] WHO. Repository of systematic reviews on interventions in environment, climate change and health [Internet]. 2024 [cited 2024 Feb 23]. https://www.who.int/publications/m/item/repository-of-systematic-reviews-on-interventions-in-environment--climate-change-and-health. 10.1186/s12940-024-01105-y PMC11495238 39434138

[5] Pollock M, Fernandes R, Becker L, Pieper D, Hartling L, Chapter V. Overviews of Reviews. Cochrane Handbook for Systematic Reviews of Interventions [Internet]. version 6.4. Cochrane; 2023 [cited 2024 Feb 23]. www.training.cochrane.org/handbook.

[6] Cochrane reviews about occupational. safety and health | Cochrane Work [Internet]. [cited 2024 Mar 8]. https://work.cochrane.org/cochrane-reviews-about-occupational-safety-and-health.

[7] Richardson WS, Wilson MC, Nishikawa J, Hayward RS. The well-built clinical question: a key to evidence-based decisions. ACP J Club. 1995;123:A12–3. 10.7326/ACPJC-1995-123-3-A12 7582737

[8] Shea BJ, Reeves BC, Wells G, Thuku M, Hamel C, Moran J, et al. AMSTAR 2: a critical appraisal tool for systematic reviews that include randomised or non-randomised studies of healthcare interventions, or both. BMJ. 2017;358:j4008. 28935701 10.1136/bmj.j4008 PMC5833365

[9] Guyatt GH, Oxman AD, Vist GE, Kunz R, Falck-Ytter Y, Alonso-Coello P, et al. GRADE: an emerging consensus on rating quality of evidence and strength of recommendations. BMJ. 2008;336:924–6. 18436948 10.1136/bmj.39489.470347.AD PMC2335261

